# Improved chromium tolerance of *Medicago sativa* by plant growth-promoting rhizobacteria (PGPR)

**DOI:** 10.1186/s43141-021-00254-8

**Published:** 2021-10-06

**Authors:** Nabil Tirry, Aziza Kouchou, Bouchra El Omari, Mohamed Ferioun, Naïma El Ghachtouli

**Affiliations:** Microbial Biotechnology and Bioactive Molecules Laboratory, Sciences and Technology Faculty, Sidi Mohamed Ben Abdellah University, Fes, Morocco

**Keywords:** Plant growth-promoting rhizobacteria, Metallic stress, *Medicago sativa*, Oxidative stress, Phytoremediation

## Abstract

**Background:**

Soil pollution by heavy metals increases the bioavailability of metals like hexavalent chromium (Cr (VI)), subsequently limiting plant growth and reducing the efficiency of phytoremediation. Plant growth-promoting rhizobacteria (PGPR) have substantial potential to enhance plant growth as well as plant tolerance to metal stress. The aim of this research was to investigate Cr (VI) phytoremediation enhancement by PGPR.

**Results:**

The results showed that the 27 rhizobacterial isolates studied were confirmed as Cr (VI)-resistant PGPR, by using classical biochemical tests (phosphate solubilization, nitrogen fixation, indole acetic acid, exopolysaccharides, hydrogen cyanide, siderophores, ammonia, cellulase, pectinase, and chitinase production) and showed variable levels of Cr (VI) resistance (300–600 mg/L). The best four selected Cr (VI)-resistant PGPR (NT15, NT19, NT20, and NT27) retained most of the PGP traits in the presence of 100–200 mg/L concentrations of Cr (VI). The inoculation of *Medicago sativa* with any of these four isolates improved the shoot and root dry weight. The NT27 isolate identified using 16S rDNA gene sequence analyses as a strain of *Pseudomonas* sp. was most effective in terms of plant growth promotion and stress level decrease. It increased shoot and root dry weights of *M. sativa* by 97.6 and 95.4%, respectively, in the presence of Cr (VI) when compared to non-inoculated control plants. It also greatly increased chlorophyll content and decreased the levels of stress markers, malondialdehyde, hydrogen peroxide, and proline. The results of the effect of *Pseudomonas* sp. on Cr content and bioaccumulation factor (BAF) of the shoots and roots of *M. sativa* plants showed the increase of plant biomass concomitantly with the increase of Cr root concentration in inoculated plants. This would lead to a higher potential of Cr (VI) phytostabilization.

**Conclusions:**

This study demonstrates that the association *M. sativa-Pseudomonas* sp. may be an efficient biological system for the bioremediation of Cr (VI)-contaminated soils.

## Background

The intensive urbanization and civilization of society are responsible for the prominent increase of rapid industrial development and the spread of metals in soils. Metal soil contamination is recognized as one of the biggest environmental concerns worldwide and constitutes a permanent threat to ecosystems, agricultural sustainability, and human health [1]. The agricultural sector suffers horribly from the increase over time of metal pollution, such as lead (Pb), cadmium (Cd), chromium (Cr), mercury (Hg), and Arsenic (As) causing a significant decrease in plant growth and crop yield [2]. Heavy metals are also used in various terrestrial chemical fungicides and fertilizers, wastewater irrigation, and sewage sludge causing heavy metal contamination of water resources and agricultural soils [2, 3].

Cr is one of the most polluting heavy metals that is commonly used in the production of electroplating, stainless steel, textile dyeing, and in the leather industry, mainly in chrome tanning of skins [4, 5]. Among the different types of Cr forms (Cr^+6^, Cr^+5^, Cr^+4^, Cr^+3^, Cr^+2^, Cr^+1^, Cr^0^, Cr^−1^, and Cr^−2^), the most stable are Cr (VI) and Cr (III). The excessive accumulation of Cr (VI) in the soil causes enormous problems for plant growth and crop productivity [6]. A higher intake of Cr (VI) slows down seedling development, germination process, and root growth [7–9]. The interference of Cr (VI) with nutrient uptake, such as phosphorus, within the intracellular membrane structures and photosynthesis, increases plant phytotoxicity. This is due to lipid peroxidation through reactive oxygen species (ROS) and modification of antioxidant activities [9, 10]. Cr crossing the plasma membrane oxidizes proteins and nucleic acid through the production of reactive oxygen species (ROS) due to its strong oxidizing nature, such as radicals, O^2−^, OH^−^, and H_2_O_2_ [7, 11]. Higher accumulation of Cr (VI) in plant tissues can affect the chlorophyll content, transpiration process, transport of electrons, CO_2_ fixation, photophosphorylation, photosynthetic enzyme activity, and stomatal conductance, which leads to a significant reduction of the photosynthetic rate [12–14].

Several efforts have been made to develop technologies useful for extracting and removing toxic heavy metals from water and soil, such as chemical oxidation or reduction, filtration, chemical precipitation, ion exchange, and electrochemical treatment [15]. However, these processes adversely affect the environment and the health of soil, plants, and humans. Also, when the concentration of heavy metals is low, these techniques are mostly ineffective and expensive [16]. Therefore, in this context, using eco-friendly approaches like plant growth-promoting rhizobacteria (PGPR)-assisted phytoremediation could be one of the best-suited choices to improve crop productivity and to alleviate heavy metals problems [17–21]. Metal hyperaccumulating plants have garnered considerable attention nowadays. *Medicago sativa* (alfalfa) for example is considered as an excellent fodder legume plant due to its high biomass productivity and its low susceptibility to environmental stresses like salinity and drought [22, 23]. It is also proposed as a promising material for metal phytoextraction [24, 25].

Numerous reports have investigated the use of PGPR to reduce efficiently Cr (VI) bioavailability and lower the Cr absorption by the plants. The main mechanisms of Cr (VI) bioremediation are biosorption (sorption of Cr (VI) by microbes and biological-based materials) and biotransformation (which convert more mobile and toxic Cr (VI) to non-toxic form Cr (III)) [26–28]. The interconnection between plants and rhizospheric microbes plays a vital role in the enhancement of phytoremediation efficacy via a mechanism called “bio-assisted phytoremediation” [29]. PGPR resistant to heavy metals have the potential to relieve heavy metal stress by improving plant development. The PGPR can similarly improve the growth and resistance of plants to Cr (VI) through mechanism of biocontrol and growth promotion. It includes phytohormones stimulation, decreased stress-induced ethylene production by synthesized enzyme ACC (1-aminocyclopropane-1-carboxylate) deaminase; production of antioxidant enzymes to scavenge ROS; production of ammonia, HCN, and siderophores; phosphate solubilization; nitrogen fixation; and bacterial secretion of extracellular polymeric substances (EPS) [28, 30–32]. Such PGPR with multiple properties of Cr resistance combined with plant growth promotion may be more essential for phytoextraction and plant growth. Thus, the present study was aimed at the isolation of Cr-resistant PGPR and the evaluation of their performance under Cr stress. Hence, pot experiments were designed to analyze the effect of selected Cr (VI)-resistant PGPR interaction with *M. sativa* species to alleviate Cr stress and to enhance Cr (VI) bioremediation.

## Methods

### Bacteria isolation

The bacteria were isolated from rhizospheric soil of various plants (alfalfa, wheat, barley) from an agricultural area (33° 56′ N, 5° 13′ W, 499 m altitude) in the Fez region, Morocco. The root system was removed along with the bulk soil from 0 to 20 cm depth, and the rhizosphere soil was recovered, placed in sterile plastic bags, transported to the laboratory on an ice pack, and kept at 4 °C until ready to be processed. The isolation of PGPR was accomplished on the basis of phosphate solubilization, which represents a substantial PGP trait. Briefly, 5 g rhizosphere soil was mixed into 45 mL distilled water. Further serial dilutions (10^−7^) were prepared from soil solution (10^−1^) with 0.9 mL distilled water [33]. An aliquot (0.1 mL) from each dilution was used to inoculate National Botanical Research Institute’s phosphate growth (NBRIP) agar plates (10 g L^−1^ d-glucose, 5 g L^−1^ Ca_3_(PO_4_), 5 MgCl_2_ 6H_2_O, 0.25 g L^−1^ MgSO_4_ H_2_O, 0.2 g L^−1^ KCl, 0.1 g L^−1^ (NH_4_)_2_SO_4_, 15 g L^−1^ agar, pH 7) that was incubated at 28 °C for 5 days [34]. The halo zones around bacterial colonies and colony morphology were used to select bacterial isolates.

### PGP traits characterization and Cr (VI) tolerance

#### PGP traits characterization

Twenty-seven bacterial isolates maintained their P-solubilization ability after three successive subcultures on the NBRIP agar medium. Their colony diameter and halo zones were recorded as described by Islam et al. [35], and their ability to solubilize inorganic phosphate was estimated as phosphate solubilization index (PSI): PSI = the ratio of the total diameter (colony + halo zone)/the colony diameter.

IAA production by the isolates was quantitatively estimated: 5 mL of LB Broth supplemented with l-tryptophan (1 g/L) and incubated at 28 ± 2 °C for 120 h with continuous shaking at 120 rpm. After centrifugation (10,000*g* for 15 min) of bacterial culture, 1 mL of the supernatant was mixed with 2 mL of Salkowski’s reagent (1.2 g FeCl_3_ 6H_2_O in 100 mL of H_2_SO_4_ 7.9 M) and incubated at room temperature for 20 min. Optical density was measured against the standard curve (serial dilutions of a solution of IAA 50 mg/mL in the LB medium) using a UV spectrophotometer at 535 nm [36].

A qualitative assay of siderophores secretion by the isolated bacteria was assessed using blue agar plates containing Chrome azurol S (CAS) (Sigma-Aldrich) with the methods prescribed by Schwyn and Neilands [37]. The positive reaction was revealed by the appearance of an orange zone around the colony, signaling siderophore production.

HCN production was determined following the Lorck [38] method. Bacterial isolates were inoculated into Lauria-Bertani plates supplied with 4.4 g/L of glycine. Sterilized filter papers (Whatman N°.1) were mounted on the top of each plate after soaking in picric acid solution (0.5% of picric acid with 2% of sodium carbonate) and incubated for 5 days at 28 ± 2 °C. The shift in the color of the filter paper from yellow to orange-red specified HCN production by bacteria.

Ammonia production was checked for the isolated bacteria on peptone water following the Cappuccino and Sherman [39] method. Bacterial isolates were inoculated into peptone water (10 mL) and incubated for 48 h at 30 ± 2 °C. Then, Nessler’s reagent (500 μL) was transferred to each tube. The shift in color of the media (development of brown to yellow color) indicated ammonia production.

Nitrogen (N_2_) fixation experience was executed, in a malate nitrogen-free mineral medium with modifications g/L (5 g malic acid, 15 g Agar, 0.5 g K_2_HPO_4_, 4 g KOH, 0.02 g CaCl_2_, 0.1 g NaCl, 0.2 g MgSO_4_ 7H_2_O, 0.024 g ZnSO_4_ 7H_2_O, 0.280 g H_3_BO_3_, 0.008 g CuSO_4_ 5H_2_O, 0.01 g FeSO_4_ 7H_2_O, 0.2 g Na_2_MoO_4_ 2H_2_O, 0.235 g MnSO_4_ 5H_2_O, and 2 mL of Bromothymol Blue (5%)) [40]. The inoculated media were incubated at 28 ± 2 °C for 3 days. Nitrogen fixation activity was regarded as positive through shifting in color from pale green to blue.

The production of EPS was tested on the modified RCV-sucrose medium [41] (yeast extract 0.1 g/L, sucrose 30 g/L, agar 15 g/L). The plates were inoculated with fresh bacterial cultures and then incubated for 5 days at 28 °C. The formation of the bacterial gel colonies on the culture medium indicates the production of EPS.

Cellulase production was tested according to Gupta et al.’s [42] protocol, using carboxymethylcellulose. Bacterial isolates were inoculated into CMC agar medium (0.05% KH_2_PO_4_, 0.025% MgSO_4_ 7H_2_O, 0.2% CMC (viscosity 10–55 cps, Aldrich Chemical Co.), 1.5% agar) and incubated for 24 h at 30 °C. The CMC plates were then flooded twice: first, for 15 min, with an aqueous solution of Congo red (1% w/v), followed by 1 M NaCl for 15 min. A transparent halo was deemed positive for cellulase production.

Pectinase production was assessed using an ammonium mineral agar medium (AMA) [43]. Bacterial isolates were inoculated into AMA medium with some modifications (apple pectin (Sigma, St. Louis, MO) (0.5%), Na_2_HP0_4_ (0.45%), KH_2_P0_4_ (0.3%), (NH_4_)_2_S0_4_ (0.2%), yeast extract (0.15%), MgS0_4_ 7H_2_0 (0.03%), (FeS0_4_ 7H_2_0 and CaC1_2_ (0.0002%)), (MnS0_4_, H_3_B0_3_, and Na_2_Mo0_4_ 2H_2_0 (0.00001%)), ZnS0_4_ 7H_2_0 (0.000017%), CuS0_4_ 8H_2_0 (0.00005%), agar (1.5%)) and incubated for 72 h at 28 ± 2 °C. Pectinase production was shown by clear halos around the colonies after flooding each plate with hexadecyltrimethyl ammonium bromide (2%).

The chitinase production was tested following Moon et al.’s [44] protocol, using 0.5% colloidal chitin agar medium (yeast extract (0.05%), MgSO_4_·H_2_O (0.05%), K_2_HPO_4_ (0.1%), MnSO_4_·H_2_O_2_ (0.001%), ZnSO_4_·7H_2_O (0.001%), FeSO_4_·7H_2_O (0.001%), agar (1.5%)). Inoculated media with bacterial isolates were incubated for 3 days at 28 ± 2 °C. A transparent halo was deemed positive for chitinase production. All assays were performed in triplicate.

#### Cr (VI) resistance of the bacterial isolates

The resistance of the isolates to Cr (VI) was assessed using the dilution plate process with a determination of the minimum inhibitory concentrations (MIC) for each bacterial isolate. For this purpose, the bacterial isolates were cultured in Petri dishes containing LB agar medium supplemented with Cr (VI) (K_2_Cr_2_O_7_) at concentrations from 0 to 1000 mg/L. The Cr solution was filter sterilized before being added to the agar medium. After 48 h of incubation at 30 °C, the minimum inhibitory concentration (MIC) was determined as the lowest concentration at which no viable colony-forming units (CFU) were observed [45].

#### Effects of Cr (VI) on the PGP traits of the selected bacteria

Four bacterial isolates were selected on the basis of PGP traits and Cr (VI) resistance and tested for their ability to maintain PGP characteristics under Cr (VI) stress. The LB medium was supplemented with varying concentrations of Cr (VI) (100, 150, and 200 mg/L), and the PGP proprieties (P solubilization, N_2_ fixation, IAA, NH_3_, HCN, cellulase, pectinase, and chitinase production) were evaluated as described above.

### Plant growth assay of *M. sativa* and tolerance to Cr (VI) exposure

#### Experimental design

Experiments were conducted in plastic pots containing soil collected from agricultural land in the Fez region. The soil of the experiments (pH 8.1, organic matter 12.93 g/kg, available phosphate 13.25 mg/kg, and available N 0.73 g/kg) was artificially contaminated with an aqueous solution of Cr (VI) (K_2_Cr_2_O_7_), to have a concentration of 10 mg of Cr (VI) per kilogram of soil.

Bacterial inoculums of each of the four selected bacteria were provided in LB medium and incubated for 24 h at 28 ± 2 °C. The cells after centrifugation (6000*g* for 20 min) were washed twice with sterile saline solution and resuspended in sterile saline solution and then diluted with sterilized water to achieve an optical density of 0.6 corresponding to 10^8^ CFU/mL.

Alfalfa seeds were surface-sterilized and germinated in soft agar plates 0.7% (w/v) water-agar. Plantlets were transplanted in the culture devices (500 g of soil into a plastic cup (10 × 9 × 20 cm)), with 3 plants per pot (3 pots for each treatment). Then, 3 mL of PGPR inoculum (DO 10^8^ CFU/mL) were added to each pot (1 mL per plant). The pot culture experiment was arranged in randomized design containing four treatments: (i) absence of bacteria and Cr (VI) (negative control), (ii) absence of bacteria and presence of Cr (VI) (positive control), (iii) presence of bacteria and absence of Cr (VI), and (iv) presence of bacteria and Cr (VI). Two days later, the pots were inoculated with 2 mL of a suspension of each bacterial culture (10^8^ CFU/mL). Two milliliters of saline solution was added to the uninoculated plants. Pots were positioned in a greenhouse (approximately 16 h photoperiod, 26–30 °C day and 18–22 °C night) and watered regularly. Five weeks later in the budding stage (from this stage through early flower is usually ideal to harvest high-quality alfalfa), plants were harvested and washed with deionized water, then divided into roots and shoots. The biomass yield was estimated after oven drying at 65 °C until constant weight.

### Plant analyses

#### Chlorophyll content

For the assessment of leaf chlorophyll content, Moran and Porath’s [46] methodology was followed. The *M. sativa* leaves (50 mg) were homogenized with acetone (80%), and the extract was centrifuged for 5 min (9000*g* at 4 °C). Then, the optical density was measured at 646.8 nm and 663.2 nm. The total chlorophyll was determined using the following equation: [(7.15 × A_663.2_) + (18.71 × A_646.8_)] *V*/*M*, where *V* is the final volume of the filtrate and *M* is the fresh weight of the leaf. It was expressed as mg/g fresh weight of leaf tissue. The chlorophyll a/b ratios were also calculated.

#### Proline content

For the assessment of proline content of the leaves, Bates et al.’s [47] methodology was followed. Plant material (0.5 g) was mashed in 10 mL of aqueous sulfosalicylic acid 3%. Then, 2 mL of filtrate was mixed with 2 mL of ninhydrine and 2 mL of glacial acetic acid. After incubation for 1 h at 100 °C, the reaction was stopped in an ice bath and 4 mL of toluene was added to each tube. Then, the optical density was measured at 525 nm. Free proline per gram of fresh weight was calculated as follows: [(μg proline/mL × mL toluene)/115.5 μg/μmole]/[(g sample)/5] = μmoles proline/g.

#### Hydrogen peroxide content

For the assessment of hydrogen peroxide (H_2_O_2_) content of the leaves, Jana and Choudhuri’s [48] methodology was followed with some modifications. One gram of leaves was homogenized with 0.1% trichloroacetic acid (TCA) (15 mL) and centrifuged for 20 min at 6000*g*. The supernatant (0.5 mL) was added to 10 mM phosphate buffer pH = 7 (0.5 mL) and 1 mM KI (1 mL). Then, the optical density was measured at 390 nm. From a standard curve prepared using known H_2_O_2_ concentrations, the sum of H_2_O_2_ was determined and expressed as mM/g fresh weight of leaf tissue.

#### Malondialdehyde content

To determine the malondialdehyde (MDA) content in plant leaves, Heath and Packer’s [49] methodology was adopted. Briefly, 0.2 g of leaves was homogenized with 5 mL of (0.5% 2-thiobarbituric acid (TBA) and 20% TCA) solution, and 1 miL of alcoholic extract was added to 1 mL of 20% TCA to prepare the control. The mixture was heated for 30 min at 95 °C, cooled at room temperature, and centrifuged (5000*g* for 10 min at 25 °C). Optical density was measured at 532 nm and 600 nm.

### Effect of bacterial inoculation on plant phytoremediation potential

This study was focused on plants inoculated by the bacterial isolate NT27 that showed interesting performance in terms of PGP traits under Cr (VI) stress, plant growth, and tolerance to Cr (VI). The plant’s phytoremediation potential was assessed by analyzing Cruptake by root and shoot tissues of plants grown as described above.

Approximately 200 mg of powdered plant tissue was digested after 24 h of drying at 70 °C [50]. Then, using the inductively coupled plasma atomic emission spectrometer (ICP-AES) (Jobin Yvon), total Cr content was determined in root and shoot tissues.

To estimate the metal uptake in plant sections, the bioaccumulation factor (BAF) was determined. It provides an index of a plant’s ability to absorb a specific metal relative to its medium concentration [51].

$$ \mathrm{BAF}\ \mathrm{root}=\mathrm{Metal}\ \mathrm{concentration}\ \mathrm{in}\ \mathrm{the}\ \mathrm{roots}\kern0.5em /\mathrm{Metal}\ \mathrm{concentration}\ \mathrm{in}\ \mathrm{the}\ \mathrm{soil} $$

$$ \mathrm{BAF}\ \mathrm{shoot}=\mathrm{Metal}\ \mathrm{concentration}\ \mathrm{in}\ \mathrm{the}\ \mathrm{shoots}/\mathrm{Metal}\ \mathrm{concentration}\ \mathrm{in}\ \mathrm{the}\ \mathrm{soil} $$

### Molecular identification

The selected isolate NT27 was characterized by a molecular identification approach using the universal primers fD1 (50 AGA GTT TGA TCC TGG CTC AG 30) and rD2 (50 ACG GCT ACC TTG TTA CGA CTT 30) [52]. Bacterial DNA extraction and fragment of rDNA amplification were realized as described by Tirry et al. [53]. The sequences obtained were checked and extracted by Mega X (version 10.0.5). Related sequences were obtained from the GenBank database, National Center for Biotechnology Information (NCBI), using the BLAST analysis, and then accession number was obtained after submission to the NCBI GenBank database. Sequences were aligned to their nearest neighbors using the MUSCLE program [54], and then a phylogenetic tree was constructed using the MEGA-X program [55].

### Statistical analysis

In order to determine the significant differences among treatments, the data collected were submitted to ANOVA analysis by using the Minitab 18 software. All the values were compared using Tukey’s method at *p* ≤ 0.05.

## Results

### PGP traits and Cr (VI) resistance of the bacterial isolates

In the present work, 27 bacterial isolates were isolated from the plant rhizosphere based on the solubilization of inorganic phosphate. These isolates showed different PGP traits (phosphate solubilization, nitrogen fixation, IAA, HCN, siderophores, ammonia, EPS, and hydrolytic enzyme production), with different degrees of tolerance to Cr (VI) (Table 1). Four isolates NT15, NT19, NT20, and NT27 were selected on the basis of their Cr (VI) tolerance and their PGP characteristics. They showed high resistance to Cr (VI) concentrations up to 600 mg/L. They also showed interesting (PGP) traits, for example, higher values of PSI (3.6) and IAA (572.27 μg/mL) were recorded by NT15 and NT20 isolates, respectively. Furthermore, the selected isolates showed other PGP traits like N_2_ fixation, EPS, NH_3_, HCN, siderophores, cellulase, pectinase, and chitinase production (Table 1).

**Table 1:** Minimum inhibitory concentrations (MIC) of Cr (VI) and plant growth-promoting (PGP) traits of the bacterial isolates. Data are the mean of replicates with *SE*±. Values with different letters are significantly different (*p* < 0.05)

Isolat	MIC (mg/L) Cr (VI)	PSI	IAA (μg/mL)	Sid	N_**2**_	EPS	NH_**3**_	HCN	Case	Pase	Chase
**NT1**	600	2.4 ± 0.2^cde^	167 ± 1.42^e^	+	–	–	+	–	+	+	+
**NT2**	400	1.33 ± 0.1^fgh^	187.5 ± 1.5^cd^	+	+	–	+	–	+	+	+
**NT3**	600	1.8 ± 0.1^cdefgh^	125.75 ± 1.45^h^	–	–	–	+	+	+	–	+
**NT4**	600	2.8 ± 0.2^bc^	10 ± 0.42^p^	+	–	–	+	+	+	+	+
**NT5**	600	2.33 ± 0.3^cdef^	71.5 ± 1.1^k^	+	–	–	+	–	+	+	+
**NT6**	600	1.14 ± 0.2^h^	144.75 ± 1.37^fg^	–	+	–	+	–	+	–	–
**NT7**	400	1.83 ± 0.3^cdefgh^	161.75 ± 1.47^e^	+	+	–	+	+	+	–	+
**NT8**	400	2 ± 0.1^cdefgh^	63.25 ± 1.18^lm^	–	–	–	+	+	–	+	+
**NT9**	600	1.25 ± 0.1^gh^	61.5 ± 1.15^lm^	+	+	+	+	+	–	+	+
**NT10**	300	2 ± 0.2^cdefgh^	92.25 ± 1.21^ij^	+	–	–	+	+	+	+	+
**NT11**	600	1.44 ± 0.3^efgh^	144 ± 1.19^g^	+	+	–	+	+	–	–	+
**NT12**	400	2 ± 0.2^cdefgh^	143.75 ± 1.22^g^	+	+	+	+	+	+	–	–
**NT13**	300	1.92 ± 0.4^cdefgh^	189.5 ± 2.43^c^	+	+	–	+	+	+	–	+
**NT14**	300	2 ± 0.1^cdefgh^	45.5 ± 1.16^o^	+	+	–	+	+	+	+	+
**NT15**	600	3.6 ± 0.5^ab^	192.95 ± 2.5^c^	+	+	–	+	+	–	–	+
**NT16**	400	1.71 ± 0.2^defgh^	73 ± 1.25^k^	+	+	+	+	+	–	+	+
**NT17**	600	1.68 ± 0.3^defgh^	124.5 ± 1.68^h^	+	–	–	+	–	–	+	+
**NT18**	400	2.06 ± 0.4^cdefgh^	96.75 ± 1.23^i^	+	–	–	+	–	–	+	+
**NT19**	600	2.55 ± 0.1^cd^	88.63 ± 1.25^j^	+	+	+	+	–	+	+	+
**NT20**	600	2.1 ± 0.1^cdefgh^	572.27 ± 4.25^a^	+	+	+	+	+	+	+	+
**NT21**	400	2.07 ± 0.4^cdefgh^	181.5 ± 1.31^d^	+	–	–	+	+	+	–	+
**NT22**	600	2.54 ± 0.2^cd^	49 ± 1.14^no^	+	+	–	+	+	–	–	–
**NT23**	300	2.57 ± 0.3^cd^	152 ± 2.24^f^	+	–	–	+	+	+	–	+
**NT24**	400	2.21 ± 0.1^cdefg^	213.25 ± 3.44^b^	+	–	–	+	+	–	+	+
**NT25**	300	3.91 ± 0.6^a^	56.25 ± 1.16^mn^	+	–	–	–	+	+	–	–
**NT26**	600	2.09 ± 0.2^cdefgh^	46.75 ± 1.17^o^	+	+	–	+	–	+	–	+
**NT27**	600	2.62 ± 0.3^cd^	64.11 ± 1.13^kl^	+	+	+	+	+	+	+	+

*+* positive test, – negative test, *Sid* siderophores, *Chase* chitinase, *Case* cellulase, *Pecase* pectinase, *PSI* phosphate solubilization index

### PGP traits of the selected bacteria under Cr (VI) stress

The ability of the selected isolates to maintain different PGP traits in the presence of Cr (VI) at concentrations ranging from 100 to 200 mg/L is presented in Table 2.

**Table 2:** Growth-promoting traits of the selected bacterial isolates (NT15, NT19, NT20, and NT 27) in the presence of Cr (VI) at 100, 150, and 200 mg/L. Data are the mean of replicates with *SE*±. Values with different letters are significantly different (*p* < 0.05)

Isolate	[Cr (VI)] (mg/L)	PGP traits							
		PSI	N_**2**_	[IAA] (mg/L)	NH_**3**_	HCN	Case	Chase	Pecase
**NT15**	Control	3.55 ± 0.5^a^	+	197.63 ± 2.43^a^	+	+	–	+	–
	100	3.04 ± 0.3^a^	+	164.74 ± 1.44^b^	+	–	–	–	–
	150	2.90 ± 0.3^a^	+	142.63 ± 1.42^c^	+	–	–	–	–
	200	–	–	47.11 ± 0.17^d^	+	–	–	–	–
**NT19**	Control	2.50 ± 0.2^a^	+	89.21 ± 1.16^a^	+	–	+	+	+
	100	2.00 ± 0.1^a^	–	71.58 ± 1.17^b^	+	–	+	–	–
	150	1.78 ± 0.0^a^	–	63.29 ± 0.41^c^	+	–	+	–	–
	200	–	–	31.71 ± 0.11^d^	+	–	+	–	–
**NT20**	Control	2.05 ± 0.4^a^	+	560 ± 5.65^a^	+	+	+	+	+
	100	1.58 ± 0.2^a^	+	353.50 ± 4.55^b^	+	–	+	–	+
	150	0.71 ± 0.1^a^	+	247.36 ± 2.51^c^	+	–	+	–	+
	200	–	+	115.44 ± 1.22^d^	+	–	+	–	–
**NT27**	Control	2.55 ± 0.3^a^	+	66.14 ± 1.17^a^	+	+	+	+	+
	100	1.44 ± 0.3^ab^	+	49.11 ± 1.12^b^	+	+	+	–	+
	150	1.12 ± 0.2^b^	+	35.26 ± 0.43^c^	+	–	+	–	+
	200	0.95 ± 0.1^b^	+	19.40 ± 0.03^d^	+	–	+	–	–

*+* positive test, *–* negative test, *Chase* chitinase, *Case*: cellulase, *Pecase* pectinase, *PSI* phosphate solubilization index

The results show that for the strain NT15, the IAA production decreased by 16.64%, 27.8%, and 76.2%, compared to the control at 100, 150, and 200 mg/L of Cr (VI), respectively. Decreases of 14.4% and 18.3% for the phosphate solubilization index compared to the control were observed at 100 and 150 mg of Cr (VI), respectively, followed by total inhibition at 200 mg/L. The nitrogen fixation was maintained until 150 mg whereas ammonia production was maintained at all concentrations of Cr (VI). HCN and chitinase production was inhibited at all concentrations of Cr (VI).

For the isolate NT19, decreases of 19.76%, 29%, and 64.45% of the IAA production, compared to the control, were obtained at 100, 150, and 200 mg/L of Cr (VI), respectively. Decreases of 20% and 28.8% in the P solubilization index, compared to the control, were observed at 100 and 150 mg/L of Cr (VI), respectively, followed by total inhibition at 200 mg/L of Cr (VI). At all concentrations studied of Cr (VI), the isolate was able to maintain NH_3_ and cellulase production but was unable to fix nitrogen and to produce pectinase and chitinase.

For the isolate NT20, decreases of 36.87%, 55.8%, and 79.4% in IAA production, compared to the control, were observed at 100, 150, and 200 mg/L of Cr (VI), respectively. Decreases of 23% and 65.4% in phosphate solubilization index, relative to the control, were observed at 100 and 150 mg/L, respectively, and a total inhibition was obtained at 200 mg/L of Cr (VI). At all concentrations of Cr (VI), the isolate retained its ability to fix nitrogen, ammonia, and cellulase production whereas HCN and chitinase productions were inhibited. Pectinase production was inhibited at the concentration of 200 mg/L of Cr (VI).

For the isolate NT27, decreases of 25.75%, 46.69%, and 70.67% in IAA production, compared to the control, were observed at 100, 150, and 200 mg/L of Cr (VI), respectively. Decreases of 43.53%, 56.1%, and 62.74% in the phosphate solubilization index, compared to the control, were observed at 100, 150, and 200 mg/L of Cr (VI), respectively. Ammonia production and nitrogen fixation were maintained at all concentrations of Cr (VI). The strain was able to produce HCN and pectinase till the concentrations of 100 and 150 mg/L, respectively, whereas its ability to produce chitinase was lost at all concentrations of Cr (VI).

### Effect of bacterial inoculation on the tolerance of *M. sativa* to Cr (VI) stress

#### Plant growth

**Fig. 1:**
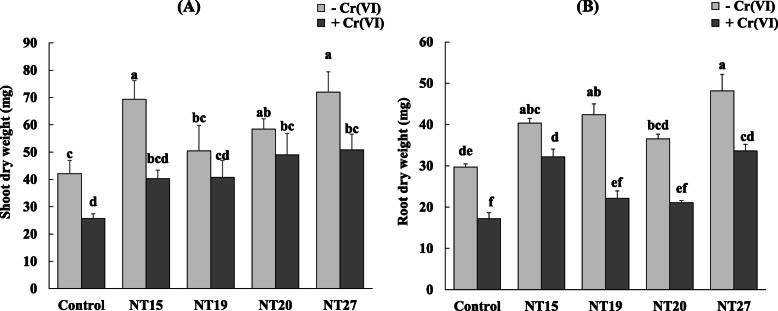
Effect of inoculation with NT19, NT15, NT20, and NT27 on the dry weight of the shoot (**A**) and root (**B**) of *M. sativa* in the absence (−Cr (VI)) and the presence of Cr (VI)(+ Cr(VI)). Values are means ± *SE*; values with different letters are significantly different (*p* < 0.05)

The effect of the four selected isolates (NT15, NT19, NT20, and NT27) on alfalfa plant growth was studied in the presence of 10 mg/L of Cr (VI) (Fig. 1a, b). In uninoculated plants (control), the results showed a reduction of 38.87% and 42.13% in the dry weight of shoots and roots, respectively, in plants subjected to Cr (VI) stress. Inoculation with bacterial cells resulted in increased plant growth both in untreated and Cr (VI)-treated plants. In the absence of Cr (VI) stress, inoculation with NT15 and NT27 significantly (*p* < 0.05) increased the dry weight of plant shoot by 64.7% and 70.9%, respectively, compared to the control. An increase in the dry weight of the roots was observed after inoculation with the bacteria, with a maximum value of 62%, observed in plants inoculated with NT27, followed by 42.7% in plants inoculated with NT19, in comparison with the uninoculated plants. In the presence of Cr (VI), inoculation with bacteria reduced the negative effect of Cr (VI) on plant growth. A maximum increase in the shoot dry weight of 97.6% and 90.36% was observed in the plants inoculated by NT27 and NT20, respectively. An increase in the dry weight of the roots was observed after inoculation with the isolates, with a maximum value of 95.38% in plants inoculated by NT27, followed by 86.84% in the plants inoculated by NT20.

#### Chlorophyll content and antioxidant system

**Fig. 2:**
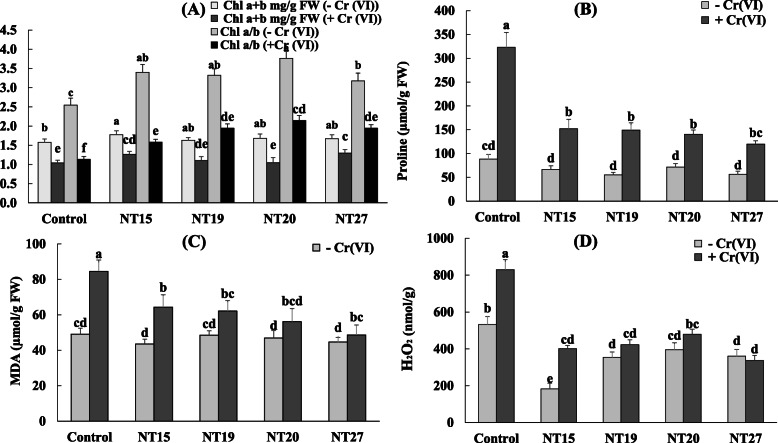
Effect of bacterial inoculation on chlorophyll (**A**), proline (**B**), MDA (**C**), and H_2_O_2_ (**D**) content of *M. sativa* leaves, in the absence (−Cr (VI)) and in the presence of (+Cr (VI)). Values are means ± *SD*; values with different letters are significantly different (*p* < 0.05)

Our results showed that the treatment of the plants with Cr (VI) caused a reduction in the total chlorophyll content and chlorophyll a/b ratio by 34% and 62.75%, respectively, in comparison with unstressed plants (Fig. 2a). In the absence of Cr (VI), all isolates increased the total chlorophyll content and chlorophyll a/b ratio of *M. sativa* leaves, with a maximum increase of total chlorophyll of 12.6%, observed in the plants inoculated with NT15 compared to uninoculated plants. In the presence of Cr (VI), the bacterial inoculation resulted in an increase of total chlorophyll levels and chlorophyll a/b ratio compared to non-inoculated stressed plants. Increases of 25% and 21.8% of total chlorophyll were observed in the plants inoculated with NT27 and NT15, respectively.

The results showed that Cr (VI) stress amplifies the accumulation of proline by 266%, MDA by 39.5%, and H_2_O_2_ by 55.86% in *M. sativa* plants (Fig.2). In the presence of Cr (VI), all the isolates significantly (*p* < 0.05) lowered the proline content in the shoots of alfalfa plants, with a maximum reduction of 63% recorded in the plants inoculated with the isolate NT27 compared to the uninoculated plants. In control plants, the decrease in the level of proline in the plants inoculated by the four isolates was not significant (Fig. 2b). Inoculation with the isolates decreased MDA values with a maximum of 42.4% observed in the plants inoculated with NT27, compared to the uninoculated plants. No significant effect of bacterial inoculation was observed in the case of non-stressed plants (Fig. 2c).

With respect to H_2_O_2_ content, a significant increase (55.86%) was observed in uninoculated plants in response to Cr (VI) stress. However, bacterial inoculation significantly reduced the accumulation of H_2_O_2_ by 51.73%, 49.1%, 42.2%, and 59.35% in plants inoculated by NT15, NT19, NT20, and NT27, respectively. In the absence of Cr (VI) stress, inoculation of plants also reduced significantly the accumulation of H_2_O_2_ in plant tissues, with a maximum decrease of 54.5% observed in plants inoculated with NT27, compared to the control (Fig. 2d).

### Effect of bacterial inoculation on metal uptake by plants

The total Cr uptake in the shoots and roots of *M. sativa* after 45 days is shown in Table 3. Data showed that the roots accumulated more Cr than shoots in both inoculated and uninoculated *M. sativa* plants. Bioaugmentation with the NT27 isolate significantly (*p* < 0.05) enhanced the root uptake of Cr and increased the bioaccumulation factor (BAF) by 49.03% as compared to uninoculated and uncontaminated control, while no significant difference was noticed in shoot Cr contents.

**Table 3:** Effect of *Pseudomonas* sp. (NT27) on Cr content (μg/g) and bioaccumulation factor (BAF) of the shoots and roots of alfalfa grown on contaminated soils with Cr (VI). Values with different letters are significantly different (*p* < 0.05)

Treatment	Chromium uptake (μg/g of dry weight)		BAF	
	Roots	Shoots	Roots	Shoots
Control	ND	ND	–	–
Cr (VI)	3.12 ± 0.3^a^	1.49 ± 0.2^a^	0.31 ± 0.1^a^	0.15 ± 0.11^a^
Cr (VI) + NT27	4.65 ± 0.33^b^	1.58 ± 0.1^a^	0.46 ± 0.1^b^	0.16 ± 0.02^a^

### Identification of the bacterial isolate

**Fig. 3:**
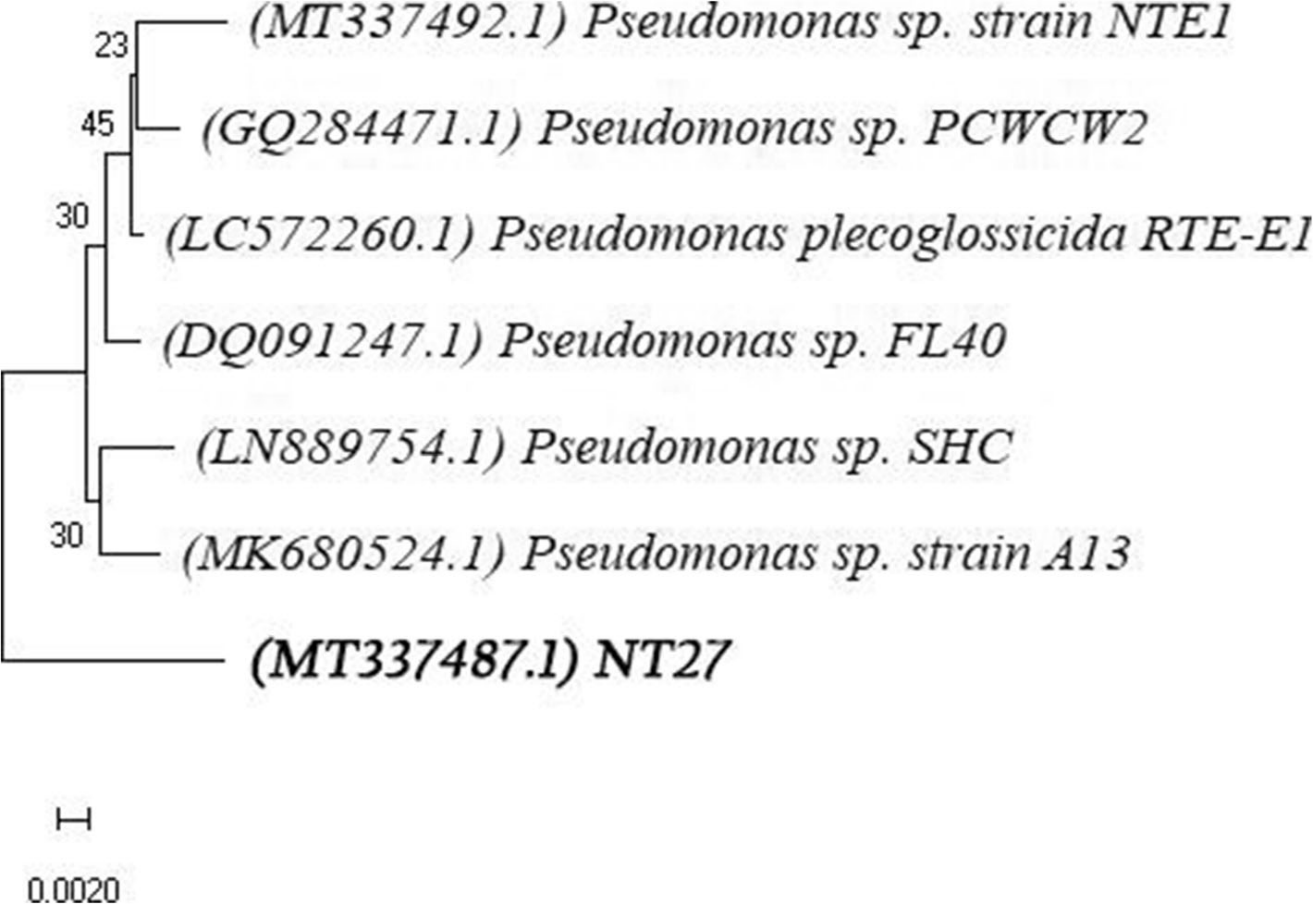
Phylogenetic analysis of the isolate NT27 based on the sequencing of the 16S ribosomal RNA gene. The scale bar indicates 0.0020 substitutions per nucleotide position

The 16S rRNA sequencing results identified the selected bacterial isolate NT27 as *Pseudomonas* sp. (GenBank: MT337487.1) which showed similarities of 99.38% with *Pseudomonas* sp. FL40 (DQ091247.1). Representative species of closely related taxa, analyzed using the neighbor-joining (NJ) algorithm, formed a *Pseudomonas* cluster consisted of the isolate NT27, *Pseudomonas* sp. strain NTE1, *Pseudomonas* sp. PCWCW2, *P. plecoglossicida* RTE-E1, *Pseudomonas* sp. FL40, *Pseudomonas* sp. SHC, and *Pseudomonas* sp. A13 (Fig. 3).

## Discussion

Cr is considered among the most toxic heavy metals because of its higher electronegativity [56, 57]. The widespread of Cr participates in the deterioration of agricultural soils on a regular basis [58, 59]. The present study was performed to isolate, screen, and characterize Cr (VI)-resistant PGPR and to determine their effects on growth and Cr (VI) toxicity tolerance of *M. sativa* plants. Our results showed that the 27 bacterial isolates studied showed various PGP properties (P solubilization, N_2_ fixation, IAA, EPS, HCN, siderophores, NH_3_, cellulase, pectinase, and chitinase production) and variable levels of Cr (VI) resistance (300–600 mg/L). Four bacterial isolates (NT15, NT19, NT20, and NT27) were selected for showing an ability to resist up to 600 mg/L of Cr (VI) concentration along with maintenance of high production of IAA, HCN, cellulase, pectinase, chitinase, and NH_3_; P solubilization; and nitrogen fixation in the presence of Cr (VI) concentrations ranging between 100 and 200 mg/L. The production of several metabolites showed a gradual decline when the concentration of Cr (VI) increases, indicating that, under stressful conditions, bacterial cells were actively involved in stress management than in other metabolic processes [60].

The detrimental effects of Cr (VI) on plant growth obtained in this study were also reported in previous works [18, 20, 61–63]. Chen et al. [64] reported that 20 mg of Cr (VI) per kilogram in soil can significantly reduce plant dry weight and root length of wheat. Also, Barcelo and Poschenrieder [65] suggested that a high accumulation of Cr (VI) in the roots and shoots restricts cell division, which limits their elongation.

After inoculation with the PGPR isolates, plant growth improved in Cr (VI)-treated *M. sativa* plants (showing similar values to uncontaminated plants both in roots and shoots) (Fig. 1). The effects of microorganism inoculation on host plant responses to Cr (VI) are poorly studied, particularly in the case of leguminous plants (*M. sativa*). Other combinations of host plant-microbes have been shown to improve plant resistance to Cr (VI). For example, Karthik et al. [66] demonstrated the positive effects of *C. funkei* on *Phaseolus vulgaris* under Cr (VI) stress. Recently, Danish et al. [67] and Gupta et al. [63] demonstrated that Cr (VI)-tolerant PGPR strains “*Agrobacterium fabrum* and *Leclercia adecarboxylata*” and “*Klebsiella* sp. CPSB4 and *Enterobacter* sp. CPSB49” respectively enhanced the growth of maize (*Zea mays*) and *Helianthus annuus* (L.) cultivated under Cr (VI) stress. Other studies have shown a positive effect of PGPR on plant growth in the presence of other heavy metals such as Cd [68], Cu and Cd [69], and Pb [70]. Rhizobacteria that promote plant growth can increase plant development and performance indirectly by reducing the toxic effects of metals or directly by producing phytohormones and growth factors [71, 72]. Indeed, PGP traits are successfully involved in promoting plant growth and attenuating the degree of toxicity in plants exposed to metal stress [72].

The high concentration of heavy metals in the soil affects plant growth because it interferes with the uptake of nutrients such as phosphorus as suggested by Halstead et al. [73]. However, this deficiency can be compensated by the ability of PGPR to solubilize phosphates which plays an important role in improving the uptake of minerals such as P by plants in metal-contaminated soils [74]. Also, the production of phytohormones by PGPR has been shown to play a key role in plant-bacteria interactions and plant growth in heavy metal-contaminated soils [75]. For instance, the stimulation of plant growth observed under Pb stress after inoculation with *P. fluorescens* has been attributed to the production of IAA [76].

Furthermore, microbial communities in the rhizospheric zone could play an important role in metal stress avoidance through secreting extracellular polymeric substances (EPS) such as polysaccharides, lipopolysaccharides, and proteins, possessing an anionic functional group that helps remove metals from the rhizosphere through the process of biosorption [28, 77]. The EPS produced by some microorganisms induce the formation of biofilms in response to the exposure to toxic heavy metals. Biofilm formation helps detoxify heavy metals by enhancing the tolerance capacity of microbial cells or by converting toxic metal ions into non-toxic forms [78]. PGPR are also characterized by the production of siderophores, which can stimulate plant growth directly under iron limitation, for example [79], or indirectly by forming stable complexes with heavy metals such as Zn, Al, Cu, and Pb and helping plants to alleviate metal stresses [80]. Indeed, siderophores have a variety of chemical structures; they have atoms rich in electrons such as electron donor atoms of oxygen or nitrogen that can bind to metal cations [81, 82]. Hannauer et al. [83] and Hernlem et al. [84] conducted a study with 16 different metals and concluded that the siderophores pyoverdin and pyochelin produced by *P. aeruginosa* are able to chelate all of these metals. In addition, siderophores secreted by PGPR can decrease free radical formation, which helps protect microbial auxins from degradation to promote plant growth [85]. Thus, in the present study, it is likely that the observed positive effect of the PGPR on plants grown under Cr (VI) might be primarily attributed to their PGP characteristics.

On the other hand, our results showed that the treatment of the plants with Cr (VI) caused a reduction in the total chlorophyll content in comparison with unstressed plants (Fig. 2a). These results are in agreement with other research reporting that the chlorophyll content decreased consistently with increasing Cr (VI) concentration [67, 86]. The alteration of chlorophyll content due to the Cr (VI) effect may be due to the inhibition of enzymes responsible for chlorophyll biosynthesis as suggested by Karthik et al. [66]. Cr (VI) toxicity inhibits photosynthesis by increasing H_2_O_2_ accumulation, superoxide production, and lipid peroxidation [87]. A higher Cr (VI) input disrupts the ultrastructure of the chloroplast and restricts the electron transport chain. Restriction of the electron transport chain diverts electrons from the PSI (electron donor side) to Cr (VI), which considerably decreases the photosynthesis rate [88, 89].

Upon inoculation by PGPR, the total chlorophyll levels increased under Cr stress. This could be due to the improvement of its synthesis or to the slowing down of the process of its degradation [66]. The improvement in chlorophyll content following inoculation could also be due to the reduction of Cr (VI) to non-toxic Cr (III) and/or to different PGP traits of these bacteria. Siderophores through the chelation reaction are known to reduce iron deficiency induced by heavy metals and thus help plants to synthesize photosynthetic compounds such as heme and chlorophyll [90, 91]. Furthermore, the enzymatic activities, phytohormones, siderophores, and organic acids of PGPR are all responsible for the reduction of toxic Cr (VI) to non-toxic Cr (III) [92–94].

Our results showed also that Cr (VI) stress amplifies the accumulation of proline in *M. sativa* plants. The increased proline content in plants has previously been identified as an adaptive response to environmental stresses [95, 96]. Proline helps plants deal with stress-related toxicity by controlling osmotic balance, detoxifying reactive oxygen species (ROS), stabilizing antioxidant enzymes, modulating gene expression, and activating multiple detoxification pathways [97, 98]. The inoculation with PGPR resulted in a substantial decrease of proline content in *M. sativa* plants, indicating that inoculated plants were less affected by Cr (VI) stress than uninoculated plants. Similar findings were found by Islam et al. [99] who reported that the level of proline in the corn plant exposed to Cr (VI) stress was significantly higher (1.08 times) than the uncontaminated plants and that inoculation with a PGPR strain reduced the proline concentration by 30%. A similar result was obtained by Karthik et al. [66], with a decrease of proline accumulation by 84.56% and 44% in the case of the association of *P. vulgaris* with two *Cellulosimicrobium* strains AR6 and AR8, respectively, under Cr (VI) toxicity.

Cr stress increased MDA content in *M. sativa* plants. MDA is a product of lipid peroxidation of the cell membrane system. MDA reacts with free amino acid groups, causing cell damage due to inter- and intramolecular reticulation of proteins [99]. The elevated MDA indicates an oxidative stress, and this may be one of the mechanisms by which Cr (VI) toxicity manifests in plant tissues. The accumulation of MDA is reported also in the case of other metallic stresses (lead, arsenic, cadmium) [100]. Plants with a low MDA content have less lipid peroxidation and, as a consequence, less oxidative damage. Upon inoculation of *M. sativa* plants by PGPR, the level of MDA decreased significantly. In accordance with our results, several works have shown the positive effect of PGPR inoculations of heavy metal-stressed plants on the attenuation of MDA levels. For example, Din et al. [32] reported that inoculation with *B. xiamenensis* PM14 significantly reduced the MDA content of *S. sesban* plant. Bruno et al. [101] also showed that inoculation of *S. bicolor* with *Bacillus cereus* TCR17, *Providencia rettgeri* TCR21, and *Myroides odoratimimus* TCR22 decreased the levels of proline and MDA in plants under Cr (VI) stress. In fact, it was already suggested that inoculation activates the protective mechanisms for ROS detoxification in plants under stress conditions, resulting in a decline in lipid peroxidation [102].

The four isolates studied in the present work (NT15, NT19, NT20, and NT27) showed a high tolerance to Cr (VI) and a high production of substances that promote plant growth in the presence of Cr (VI), demonstrating their potential to contribute to beneficial plant-microbe interactions in soils contaminated by heavy metals. More specifically, the NT27 isolate showed significant resistance to Cr (VI), characteristics of promoting plant growth and a capacity to enhance the tolerance of *M. sativa* to Cr (VI). This isolate was identified as a strain of *Pseudomonas* sp. by 16S rDNA sequence analysis. Its effect on Cr (VI) content and bioaccumulation factor (BAF) of the shoots and roots of *M. sativa* plants was significantly (*p* < 0.05) higher in comparison with uninoculated and uncontaminated control. Several works reported the increased metal concentrations in tissues of inoculated plants by the *Pseudomonas* genus. For instance, Kamran et al. [103] and Ma et al. [104] observed that *P. putida* and *Pseudomonas* sp. A3R3 increased the Cr (VI) and Ni uptake in *Eruca sativa* and *Alyssum serpyllifolium* plants, respectively. For other bacterial geniuses, Din et al. [32] and Tirry et al. [105] noticed an increased Cr (VI) accumulation in *Sesbania sesban* and *M. sativa* by the addition of *B. xiamenensis* PM14 and *Cellulosimicrobium* sp., respectively.

Nevertheless, for certain cases, it has been documented that inoculating plants with metal-resistant bacteria reduced metal uptake and increased plant biomass [106], which can be explained by the metal immobilization in the rhizosphere. In fact, several authors recorded lower Cr (VI) accumulation in bacterial inoculated plants due to the bacterial immobilization of Cr (VI) through several mechanisms, including reduction, adsorption, accumulation, and production of cell surface-related polysaccharides and proteins [107, 108]. In the present study, it is outstanding that Cr accumulation by roots was more significant than by shoots following NT27 inoculation of *M. sativa*. This is probably due to Cr (VI) reduction to Cr (III), which would have favored the immobilization of Cr (VI) in the rhizosphere and its phytostabilization in the plant roots. The phytoremediation ability of *M. sativa* plants seems to be largely favored by the strain of PGPR involved.

## Conclusions

We can conclude from our results that the inoculation of *M. sativa* species by PGPR overcoming the negative effects of Cr (VI) stress and increased the plant growth rate and the content of chlorophyll. It also greatly decreased the levels of stress markers, malondialdehyde, hydrogen peroxide, and proline.

The bacterial strains NT15, NT19, NT20, and NT27 exhibited high tolerance to Cr (VI) and produced substances favoring the growth of plants, both in normal and under Cr (VI) stress conditions, demonstrating their potential to contribute to beneficial plant-micro-organism interactions in soils contaminated by metals. This study provides clear evidence of the response of bacterial strains in the rhizosphere to Cr (VI) and the enhancement of *M. sativa* growth and antioxidant system under stress by Cr (VI). The results showed also that an enhanced Cr (VI) phytoremediation of *M. sativa* can be achieved by *Pseudomonas* sp. inoculation. Therefore, inoculation of these bacterial strains from the rhizosphere might be a good choice for application in microorganism-assisted phytoremediation approaches for the remediation of heavy metal-contaminated soils. These strains can also act as a lasting factor in the phytostabilization of Cr (VI) and a control of its entry into the food chain.

## Data Availability

Not applicable.

## Abbreviations

PGPR: Plant growth-promoting bacteria
N_2_: Nitrogen
IAA: Indole acetic acid
EPS: Exopolysaccharides
HCN: Hydrogen cyanide
BAF: Bioaccumulation factor
PSI: Phosphate solubilization index
